# Toxoplasmosis: Experimental Vaginal Infection in NMRI Mice and Its Effect on Uterin, Placenta and Fetus Tissues

**DOI:** 10.5812/ircmj.11427

**Published:** 2013-07-05

**Authors:** Parvin Dokt Bayat, Zahra Eslamirad, Saeedeh Shojaee

**Affiliations:** 1 Department of Anatomy, Arak University of Medical Sciences, Arak, IR Iran; 2 Department of Parasitology and Mycology, Arak University of Medical Sciences, Arak, IR Iran; 3 Department of Medical Parasitology and Mycology, Tehran University of Medical Sciences, Tehran, IR Iran

**Keywords:** Infection, Toxoplasma, Vaginal, NMRI Mice

## Abstract

**Background:**

*Toxoplasma gondii* is an important zoonotic pathogen. Vertical transmission of the parasite occurs when females were infected primarily during gestation. This parasite is transmitted to the fetus through the placenta and may cause miscarriage, permanent neurological damage, premature birth and visual impairment. It has been found that mouse is susceptible to Toxoplasma and is particularly an interesting model to the study of congenital infection but whether the entry of *T. gondii* through vagina route is involved in transmission of the parasite to the placenta and fetus or not.

**Objectives:**

The current study aimed to find a route of infection which perhaps carried the parasite under natural conditions in human.

**Materials and Methods:**

In the current experimental study, two 6-8 week NMRI female mice were crossed with one male. The pregnant mice were divided into 2 groups: experimental group that was infected by parasite via intra-vaginal (IV) and control group that received the same volume of normal saline via IV. One mouse from each group was killed on the fifth day after infection. The peritoneal fluid, ovary and uterus of mouse samples were taken and divided into two parts. One part used for DNA extraction and the other was kept in formalin and sent for histological study. These steps were repeated seven times and at least 10 mice in each group (case and control) were studied by molecular and histological methods.

**Results:**

PCR using DNA extracted from the experimental group showed that the parasite existed in tissues of the uterus and placenta but not in the embryos and peritoneal fluid. PCR using DNA extracted from the control group was negative.

**Conclusions:**

Tachyzoite of Toxoplasma and DNA of this parasite were observed in sub mucosa and muscles of the uterus and in the villis of placenta, but not in histological sections of the fetus. Therefore, histological and molecular results were consistent.

## 1. Background

Toxoplasmosis is a parasitic disease caused by the protozoan *Toxoplasma gondii*. *T. gondii* is an important zoonotic pathogen and has worldwide distribution (1). This disease may result a spectrum of consequences including severe congenital defects, blindness or death (1). Ingesting tissue cysts from undercooked meat, drinking drinks, contaminated with oocysts, or accidentally ingesting oocysts from the environment are the ways for human infection. Although most infections with *T. gondii* in humans are asymptomatic but in patients such as those with acquired immunodeficiency syndrome, toxoplasmic encephalitis can be life threatening (1). Vertical transmission of the parasite occurs when females are infected primarily during gestation. At this time parasite can be transmitted to the fetus through the placenta and may cause miscarriage, permanent neurological damage, premature birth and visual impairment (2). Severe cases of congenital toxoplasmosis occurs with greater frequency when the mother is infected during the first two trimesters of pregnancy, when it is acquired later, symptoms tend to be subclinical or even lacking in the fetus (3). In recent years numerous animal models have been used to study the pathology of toxoplasmosis, efficacy of vaccines, new drugs for treatment of congenital transmission, chorioretinitis and toxoplasmic encephalitis. It has been found that mouse is susceptible to *T. gondii* and is particularly an interesting model to the study of congenital infection (4). Roberts (1992) demonstrated that vertical Toxoplasma transmission only occurs in Balb/c mice infected with *T. gondii* for the first time during pregnancy (5). Arantes and Lopes studied artificial insemination of female dogs and rams respectively with *T. gondii* -positive seminal samples. Their results suggested that *T. gondii* may be sexually transmitted in domestic dogs and rams (6, 7). It has been proven that *T. gondii* penetrates in to the bloodstream and spreads within the host, but direct entry of the parasite through mucosa into genital organs has not been studied. Therefore the current study evaluated, whether the entry of *T. gondii* through vagina route is involved in direct crossing of the parasite into the uterine tube, uterus tissue and subsequent placenta and fetus or not. Then experimental animals were infected through vaginal route and the likelihood of direct transmission of parasite to the female genital tissue and placenta and fetus were studied.

## 2. Objectives

Vaginal route was selected because it was desirable to select a route of infection which perhaps carried out under natural conditions in human.

## 3. Materials and Methods

### 3.1. Parasite

In the current experimental study, the RH strain of *Toxoplasma gondii* was kindly donated to us by Department of Parasitology, Faculty of Health, Tehran Medical Sciences University. Tachyzoites were maintained by serial intraperitoneal passage in young white mouse. Under this condition the mice were ill or dead after 3 to 5 days post infection (pi). Then, peritoneal fluid was collected. Tachyzoites were counted in a Neubauer chamber. These parasites were used for infection of experimental animals via intra-vaginal (IV).

### 3.2. Mice

All procedures in this study were conducted according to international regulations for animal experimentation and approved by the Institutional Medical Ethics Committee of Arak University of Medical Sciences, Arak, Iran (Accreditation No.90-109-12, July 2011). Passage of the parasite was done on young Balb/c mice. Vaginal infection was done on 6-8 week NMRI female mice (Pasteur Institute, Iran). All these mice were housed one per cage in an air-conditioned animal room at an ambient temperature of 23 °C, relative humidity ranging from 55 ± 60% , in a 12-h (on/off light) cycle and with free access to food and water. Two female mice were crossed with one male. Visible of vaginal plug designated as zero day of pregnancy (E0). The pregnant mice were divided into 2 groups: experimental group that infected by parasite via IV and control group that received the same volume of normal saline via IV. These steps were repeated fourteen times and at least 10 mice in each group (case and control) were studied by molecular and histological methods.

### 3.3. Procedure of Infecting Animals

The number of parasites per microliter (µl) of suspension of parasite was counted. The mice were inoculated with 2 million parasites by the vagina. It should be noted that in the current study, different concentrations of parasites in identical volume of suspension were used (20 µl). Thus the parasite suspension to a final volume of 20 microliters were used for each mouse, due to the fact that increasing the volume of suspension(more than 20 µl) may lead to parasite penetration into the peritoneal cavity and increase the likelihood of parasite penetration into peritoneal cavity.

### 3.4. Inoculation Procedure in Brief

Twenty microliters of suspension containing 2 million parasites were prepared. Mouse was kept by a person in the correct position. The suspension was inoculated to vaginal canal of female mice by a liquid handling instrument on the 14th day of pregnancy.

### 3.5. Sampling

Sampling began 5 days post infection. One mouse from each group was killed on the fifth day after infection. The peritoneal fluid samples were taken before the death. Ovary and uterus of mice were isolated and divided into two parts. One part used for DNA extraction and the other were kept in formalin and sent for histological study. This experiment was repeated seven times.

### 3.6. Tissue Preparation for Light Microscopy (LM)

Uterine tissue (5mm × 5mm), placenta and embryos (total) were fixed in 10% formalin for 48 h. The tissue was briefly washed with saline, and then this tissue passed through a series of ascending concentration of ethanol and rinsing with xylene. The samples were embedded in melted paraffin and the slices with diameter of 3-µm were produced by microtome. The sections from each paraffin block were stained with Hematoxylin and Eosin (H&E) (8). Finally, sections were studied and were photographed with light microscopy (Nikon, Japan). All chemicals were purchased from Merck (Co. Germany).

### 3.7. DNA Extraction

Genomic DNA was extracted by utilizing an extraction DNA kit according to manufacturer’s instructions from blood and tissue samples (RTP Bacteria DNA Mini Kit, steratec). This product was confirmed by electrophoresis on a 0.8% agarose gel.

### 3.8. Polymerase Chain Reaction (PCR)

Standard primers TOX4 and TOX5 were selected for polymerase chain reaction (PCR) (9).

TOX4 (5´ -CGCTGCAGGGAGGAAGACGAAAGTTG-3´)

TOX5 (5´-CGCTGCAGACACAGTGCATCTGGATT-3´)

After optimization of the PCR reaction for pH and MgCl_2_ concentration, the PCR reaction was performed in a 25 µl reaction mixture containing 0.5 mM of each primer, 100 mMdNTP (Pharmacia Biotech), 60 mMTris-HCl (pH 9.0), 15 mM (NH_4_)_2_SO_4_, 2 mM MgCl_2_, 0.5 U SuperTaq (Sphaero Q). Amplification was performed on a Eppendorof thermocycler by 5 min incubation at 94 ºC, followed by 35 cycles of 30 min at 94 ºC, 30 min at 58 ºC, 30 min at 72 ºC and a final 10 min incubation at 72 ºC. Accordingly, a 529 bp fragment was constructed. This product was confirmed by electrophoresis on a 0.8% agarose gel.

## 4. Results

### 4.1. PCR Results

**Figure 1.**
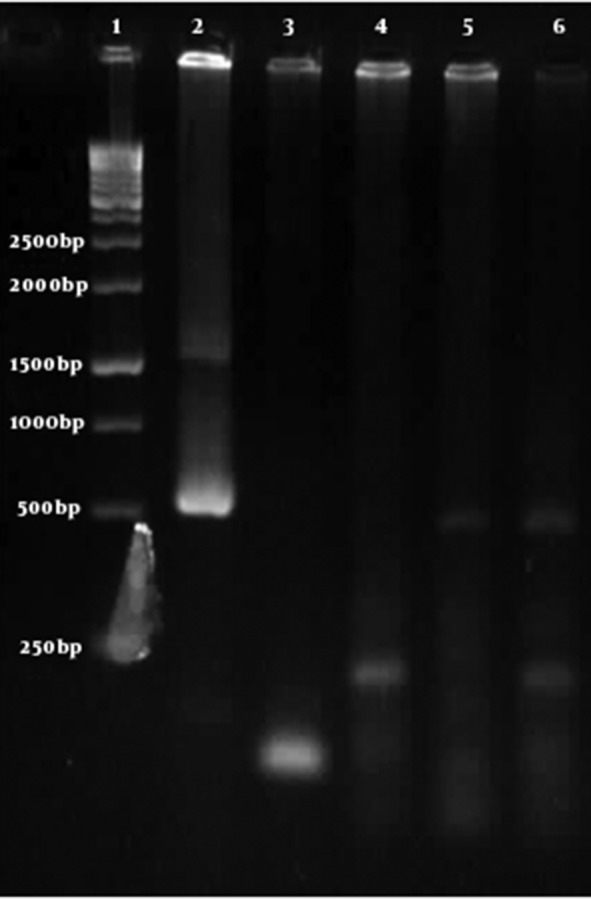
Detection of Toxoplasma DNA by PCR in Experimental Group. Lane 1: 1 Kb Molecular Size Marker, Lane 2: Positive Control with 529 bp Band, Lanes 3 and 4: Peritoneal Fluid and Fetus Tissue (Respectively) Without Expected Band, Lanes 5 and 6: Uterine and Placental Tissue (respectively) with Expected Band (529 bp).

PCR, using extracted DNA from uterine and placenta of experimental group showed an expected band (estimated 529 bp). But this band was not observed in PCR reactions using extracted DNA from peritoneal fluid and embryos of the same group. In other words, it seems that the parasite existed in tissues of the uterus and placenta but not in the embryos and peritoneal fluid (Figure 1). PCR using DNA extracted from the control group was negative. 

### 4.2. Histologic Results

**Figure 2.**
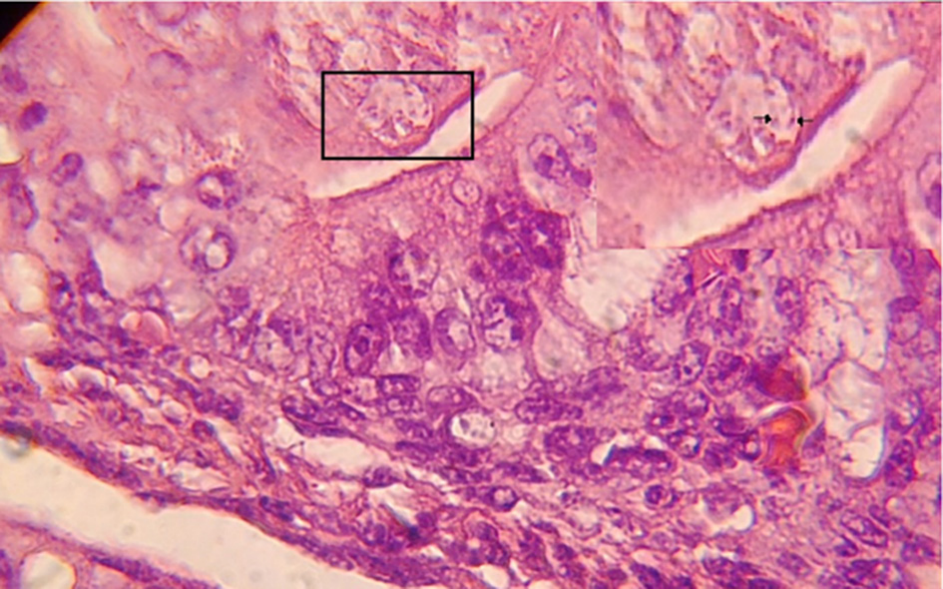
The Polymorphic Inflammatory Infiltration with Some Apoptic Sites, Congestion of Vessels in both Endometrium and Myometrium of Uterus and Parasite in Tissue of Infected NMRI mice (H&E, ×100). Upper Right Angle Shows Toxoplasma Taxchyzoite Specific

In the experimental group embryos were dead, although they were alive in control group. Evaluating by light microscope showed that normal saline treatment did not cause any tissue changes in the sections of control group which received it. But in the experimental group which received parasite, the parasite was observed in tissue, also the polymorphic inflammatory infiltration with some apoptic sites ,and congestion of vessels in both endometrium and myometrium of uterus was observed too (Figure 2). 

**Figure 3.**
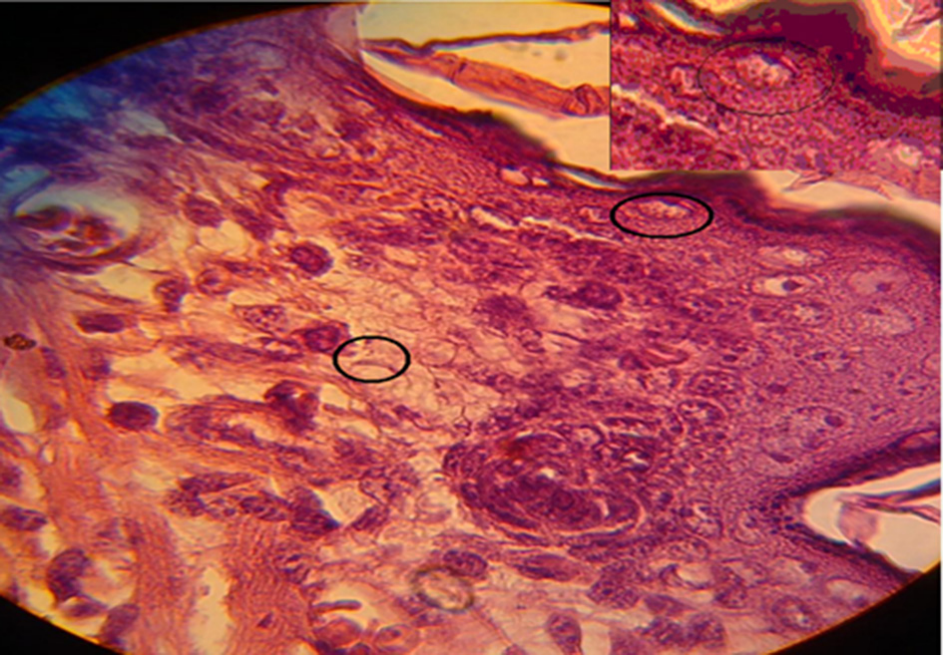
Tachyzoite of Toxoplasma gondii in Sub-mucous and Myometrium of Infected NMRI Mice (H&E × 100).Upper right Angle Shows Toxoplasma Tachyzoite Specific.

Furthermore, in sub-mucous and myometrium, tachyzoite of *Toxoplasma gondii *were observed (Figure 3). 

**Figure 4.**
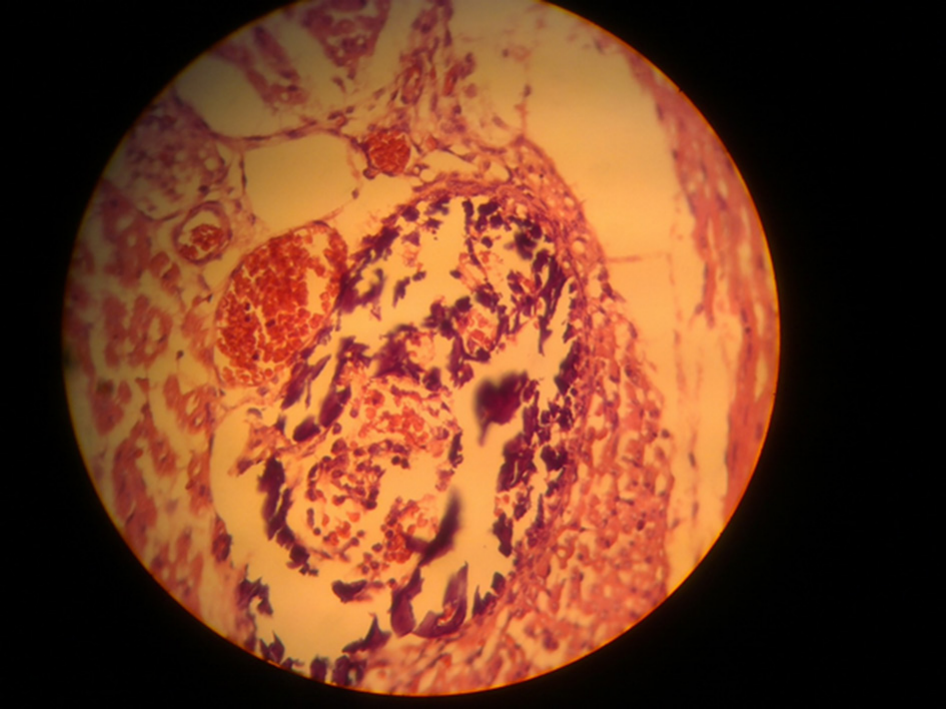
The Villi of Placenta With Vessels Congestion and Polymorphic Inflammatory Infiltration (H&E × 100).

In the same group, in the villi of placenta vessels, congestion and polymorphic inflammatory infiltration was observed (Figure 4). 

However, the parasite was not detected in fetal tissue sections and no changes were found in fetus tissues. It is noteworthy that in peritoneal fluid samples the parasite was not detected.

## 5. Discussion

Primary maternal infection during pregnancy is responsible for almost all fetal infection and its frequency depends on human seroprevalence rates that range from 10 to 80% across nations (10). The mechanisms by which *T. gondii* infects the placenta and crosses from mother to fetus are poorly understood and experimentally complicated by cross-species anatomical diversity of the placenta. Placenta is a strong regulator organ between fetus trophoblast and mother’s cell and tissue with which they come into contact (11). Identification of major site of *T. gondii* invasion in placenta may improve our understanding about barrier structure of the placenta, as well as enhancing our understanding about how *T. gondii* disseminates in fetus (11). The current study tried to answer the question that whether Toxoplasma enter the host body through the vagina and can be directly transferred to internal genital tissue, and finally can be transmitted to the placenta and fetus. Vaginal route was selected because it was desirable to select a route of infection which perhaps carried the parasite under natural conditions in human. Pregnant females in their late 2nd trimester were selected because placenta and villi are completely developed in this period. Two technical approaches were used to confirm parasite existence in histological sections of the genital system of the pregnant mouse and the PCR. Finally, results were compared. Tachyzoite of Toxoplasma was observed in submucosa and muscles of the uterus and the villis of placenta but was not observed in histological sections of the fetus. Also, parasite DNA was detected in the uterus and placenta, but not in the fetus. Therefore, histological and molecular results were consistent.

Pezerico (2009) reported that 5 to 7 days after inoculation of Toxoplasma to pregnant mice, uterus and placenta were not infected using PCR (12). In contrast, Thouvenin (1997) reported that 7 days post infection placenta was infected (13). Shiono (2007) reported that C57BL/6 mice infected with parasite on day 11 of gestation and examination on 7 days post infection showed high numbers of parasites in sections of the uterus and placenta tissues (14). When the infection was performed at a later stage of gestation (second week), parasitemia in the placenta was higher than that of observed in the early stage (day 7) of gestation (14).The results of the current study using PCR to assess the sub-mucousa and muscles of uterus and using histological sections indicated that mice infected with parasite on day 14 of gestation and examined at 5 days post infection showed parasites in the uterus and placenta. Considering the above studies shows that although the strain of the parasite and mice, and age of gestation were different but results were similar. Bittencourt described anatomical placental changes during toxoplasmosis. The most important change was the focal villitis (15). Results of the current study also confirmed the same kind of tissue changes. Pathological changes are more common and severe in the placenta than in the fetus, and placental damage is probably the primary cause of fetal death (16). Results of previous studies showed that pregnant mice were infected to toxoplasmosis through the blood but result of the current study showed that the pregnant mice may be infected to toxoplasmosis through mucous of vagina. In the present study, *Toxoplasma gondii* were detected in sub-mucousa and muscular layer of uterus. Therefore, it is possible that parasite enters directly into uterus. Also no parasites were observed in the fetus, therefore it seems that placenta is a good barrier to prevent transmission of the parasite to the fetus, but it remains a question that why fetus are dead.

Croy (2003) reported that in infected Swiss-Webster mice the factor possibly involved in resorption is spiral artery vasodilatation. IFN-γ is known to regulate spiral artery dilation in non-infectious setting (17). Also in current study we observed spiral artery vasodilatation, therefore IFN-γ as the factor may involve in fetal death. An important question remains and that is, Toxoplasma after vaginal insemination has directly entered into placenta or after entering the blood of the mother has penetrated into decidua and then into placenta. In conclusion, NMRI mice present a satisfactory model for research on congenital toxoplasmosis. So far, the study on direct passage of Toxoplasma through the mucosal tissues has not been performed. The current study showed that this parasite possibly can enter into tissue. However, it is recommended that a specific staining method be designed for *Toxoplasma gondii* in tissue. Thus differentiation of parasite from surrounding tissue is done better and more accurately.
